# AfuPmV-1-Infected *Aspergillus fumigatus* Is More Susceptible to Stress Than Virus-Free Fungus

**DOI:** 10.3390/jof9070750

**Published:** 2023-07-15

**Authors:** Gabriele Sass, Marife Martinez, Ioly Kotta-Loizou, David Stevens

**Affiliations:** 1 California Institute for Medical Research, San Jose, CA 95128, USA; mmartinez@cimr.org (M.M.); stevens@stanford.edu (D.S.); 2 Department of Life Sciences, Faculty of Natural Sciences, Imperial College London, South Kensington Campus, London SW72AZ, UK; i.kotta-loizou13@imperial.ac.uk; 3 Department of Clinical, Pharmaceutical and Biological Science, School of Life and Medical Sciences, University of Hertfordshire, College Lane Campus, Hatfield AL109AB, UK; 4 Division of Infectious Diseases and Geographic Medicine, Department of Medicine, Stanford University School of Medicine, Stanford, CA 94305, USA

**Keywords:** *Aspergillus fumigatus*, polymycovirus, fungal growth, temperature stress, cell wall stress, oxidative stress

## Abstract

Infection with Aspergillus fumigatus polymycovirus 1 (AfuPmV-1) affects *Aspergillus fumigatus* Af293’s growth in vitro, iron metabolism, resistance in intermicrobial competition with *Pseudomonas aeruginosa*, resistance to osmotic stress, and resistance to the chitin synthase inhibitor nikkomycin Z. Here, we show that response to high temperature, Congo Red-induced stress, and hydrogen peroxide are also dependent on the viral infection status of *A. fumigatus*. AfuPmV-1- infected Af293 was more susceptible than virus-free Af293 to growth inhibition by high temperature, hydrogen peroxide, Congo Red exposure, and nutrient restriction. Increased resistance of virus-free fungus was observed when cultures were started from conidia but, in the case of high temperature and hydrogen peroxide, not when cultures were started from hyphae. This indicates that the virus impairs the stress response during the growth phase of germination of conidia and development into hyphae. In conclusion, our work indicates that AfuPmV-1 infection in *A. fumigatus* impairs host responses to stress, as shown by exposure to high temperature, oxidative stress such as hydrogen peroxide, and some cell wall stresses, as shown by exposure to Congo Red (in agreement with our previous observations using nikkomycin Z) and nutrient restriction.

## 1. Introduction

*Aspergillus fumigatus* is a common fungal pathogen that often co-exists and interacts with bacterial pathogens, such as *Pseudomonas aeruginosa,* in the airways of immunocompromised patients and individuals with cystic fibrosis, where these pathogens cause acute and chronic illnesses. As an environmental resident and as a pathogen, *A. fumigatus* needs to be tolerant to a variety of adverse conditions, such as temperature changes or changes in substrates. Such changes may stem from arbitrary environmental conditions *A. fumigatus* is exposed to, or could be targeted attacks against the fungus through the increased body temperatures of infected individuals and the generation of oxidants by lung macrophages.

The fully sequenced *Aspergillus fumigatus* laboratory strain Af293 [1] is naturally infected with Aspergillus fumigatus polymycovirus (AfuPmV-1), a member of the *Polymycoviridae* [2]. AfuPmV-1 does not form conventional virions and has four double-stranded (ds) RNA genomic components, each encoding one protein, including the RNA dependent RNA polymerase responsible for the replications [3]. Closely related viruses in terms of electrophoretic profile and sequence have been reported in clinical and environmental *A. fumigatus* isolates [4,5,6], while the variant AfuPmV-1M was also found in Af293 but has an additional genomic component that encodes a polypeptide with no similarity to proteins in public databases [7].

Using naturally infected Af293, cured Af293 (virus-free), and AfuPmV-1-reinfected versions [3], we were recently able to show that a variety of stresses predominantly affect AfuPmV-1-infected versions of Af293 with respect to fungal physiology, metabolism, and response to drugs. Culture filtrates from *P. aeruginosa*, predominantly under low-iron conditions, inhibited infected Af293 to a greater extent than virus-free Af293 [8]. Timing of the production of internal and external fungal siderophores was related to the infection status of the fungus [9]. Virus-free fungus showed decreased susceptibility to *P. aeruginosa* volatiles [8], where the mechanism of action is related to production of small organic *P. aeruginosa* molecules, but unrelated to iron stress [10]. Additionally, we found that response to high salt stress was also impaired in virus-infected strains, compared to the isogenic virus-free strain [11], as previously reported for AfuPmV-1M [7]. Another study showed that virus-free fungus was less susceptible to the chitin synthase inhibitor nikkomycin Z (NikZ) than AfuPmV-1-infected fungus, whereas the anti-fungal effects of amphotericin B (AmB), voriconazole (VCZ), micafungin (MICA), and caspofungin (CASPO) were similar [12].

In the present study, we compared the effects of stress, induced by hydrogen peroxide, high temperature, Congo Red (CR), or nutrient starvation, on isogenic virus-free and AfuPmV-1-infected fungus.

## 2. Materials and Methods

### 2.1. Materials

Sabouraud Dextrose Agar (SDA) and six mm paper disks were purchased from Beckton Dickinson (Sparks, MD, USA). Bacto agar was obtained from Carolina Biological Supply Co., Burlington, NC. For all studies, 100 mm diameter plastic Petri dishes were used (E&K Scientific, Santa Clara, CA, USA). Congo Red (CR), hydrogen peroxide, and RPMI 1640 medium were purchased from Sigma-Aldrich (St. Louis, MO, USA).

### 2.2. Preparation of Agar Plates

SDA was prepared according to the manufacturer’s instructions. CR was added to SDA prior to autoclaving. H_2_O_2_ was added after autoclaving at concentrations detailed in the Results and Figure Legends. RPMI agar plates were prepared by adding 5 g Bacto agar to 100 mL distilled water. Following autoclaving, agar was mixed with 350 mL prewarmed RPMI-1640 medium, and, where indicated, with hydrogen peroxide at different concentrations. Agar plates were prepared by distributing 20 mL of liquid into Petri dishes. Plates were allowed to cool to room temperature before use. Plates were kept from light before and during the study. Plates containing H_2_O_2_ were used on the day of preparation.

### 2.3. Strains and Isolates

The use of all microbes in our laboratory was approved by the CIMR Biological Use Committee (approval no. 001-03Yr.17). Assays were performed using *A. fumigatus* isolates shown in Table 1.

The naturally infected Af293 strains 18–95 (UK) and 10–53 (USA) had been maintained on separate continents for over 10 years. The naturally infected Af293 strain 18–95 was cured from AfuPmV-1 using the protein synthesis inhibitor cycloheximide [3], resulting in strain 18–42. Purified AfuPmV-1 was re-introduced in the virus-free *Aspergillus* 18–42 by protoplast transfection [3], producing the reinfected strain 19–40. The presence or absence of AfuPmV-1 was confirmed using Northern blotting and RT-qPCR as previously described [3]. Strain 19–40 was included in comparative studies as a control for any ‘collateral damage’ from the process of curing.

### 2.4. Measuring Growth from A. fumigatus Conidia-Based Cultures on Agar, Visualized in Figure 1

Sterile paper discs were placed on 10 cm agar plates (one disk per plate) and inoculated with 10 µL of *A. fumigatus* suspensions in RPMI (10^4^ to 10^7^ conidia/mL, as detailed in the Results and Figure Legends). Colony diameters were measured after incubation at temperatures ranging from room temperature (22 °C) to 46 °C for 24 to 96 h, as detailed in the Results and Figure Legends. Areas of fungal growth were calculated using the formula area = r (radius)^2^ × π and are provided in mm^2^. Fungal growth on non-supplemented SDA or RPMI agar plates at 37 °C was regarded as 100%. Growth of each strain in the presence of treatment was normalized to that.

**Figure 1:**
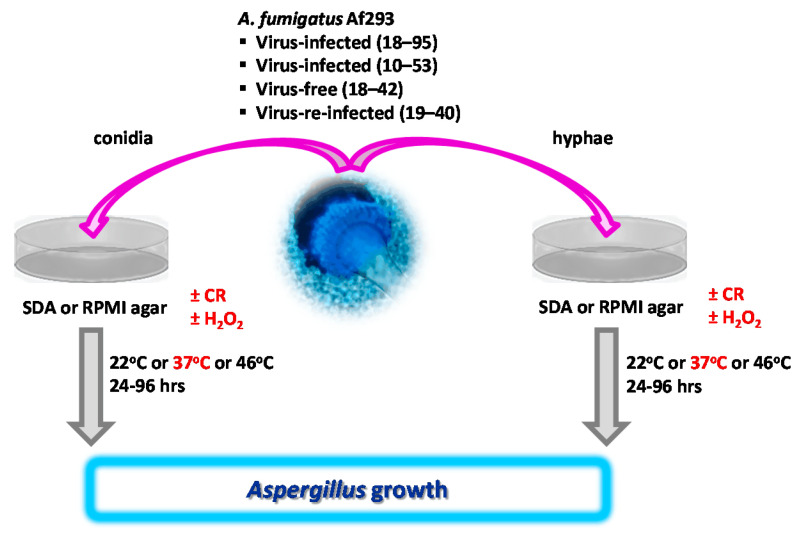
Overview of growth assays, based on conidia or hyphae. *A. fumigatus* conidia or hyphae were placed on SDA or RPMI agar plates, containing Congo Red (CR), hydrogen peroxide (H_2_O_2_), or no additives. Growth with additives was compared to growth with additives after incubation at 37 °C (red). In other experiments, growth at 37 °C was compared to growth at 22 °C or 46 °C. For details, please see the respective Methods parts.

### 2.5. Measuring Growth from A. fumigatus Hyphae-Based Cultures on Agar, Visualized in Figure 1

Sterile paper discs were placed on 10 cm SDA plates (one disk per plate) and inoculated with 10 µL of *A. fumigatus* suspensions in RPMI (10^7^ conidia/mL). Colonies were allowed to form for 48 h at 37 °C. Hyphae from the outer, conidia-free zone of a colony were harvested with the use of a sterile 6 mm stencil. The agar block was placed 1 cm from the outer edge of a fresh 10 cm agar plate, so that the hyphae-carrying part of the block faced towards the center of the plate, to allow the most space for growth. Plates were incubated at various temperatures or conditions as indicated in the Results, and in the Figure Legends. Colony radii were measured from the origin of growth to the outer periphery of growth. Areas of fungal growth were calculated using the formula area = r (radius)^2^ × π and are provided in [mm^2^]. Fungal growth on non-supplemented SDA or RPMI agar plates at 37 °C was regarded as 100%. Growth of each strain in the presence of treatment was normalized to that.

### 2.6. Temperature Treatment of Conidia

Suspensions of conidia in RPMI were prepared (10^7^ conidia/mL), and exposed to 50 °C in a heat block for 30 min, or were frozen at −20 °C or −80 °C for 24 h. Pre-treated conidia suspensions were allowed to equilibrate to room temperature before 10 µL was applied to SDA plates, and incubated at 37 °C for 48 h.

### 2.7. Statistical Analysis

Results were analyzed using Student’s *t* test. Data in this study are expressed as individual values and as their mean, where possible ± SD. Data are also reported as the percent of control, with the control for each fungal strain defined as its growth at 37 °C on agar without treatment. Each experiment in this study was performed at least twice, with a minimum of three technical replicates in each experiment. Representative experiments are shown.

## 3. Results

### 3.1. Virus-Free A. fumigatus Af293 Grows Faster Than AfuPmV-1-Infected Fungus at 37 °C

**Figure 2:**
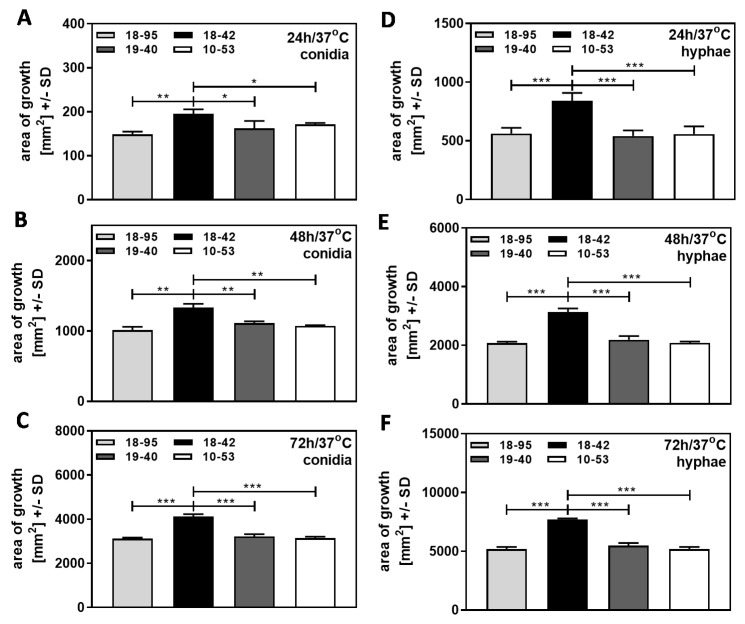
Virus-free *A. fumigatus* Af293 grows better than AfuPmV-1-infected fungus at 37 °C. AfuPmV-1-infected *A. fumigatus* Af293 (18–95, 10–53), virus-free Af293 (18–42), and reinfected Af293 (19–40) were placed on SDA plates at 10^4^ conidia/plate (**A**–**C**), or, in the form of hyphae, derived from SDA plates at 48 h of incubation (**D**,**E**) and incubated at 37 °C for 24 (**A**,**D**), 48 (**B**,**E**), or 72 h (**C**,**F**). Areas of growth were determined in mm^2^. Comparisons as indicated by the ends of the brackets. Statistical analysis: unpaired *t*-test, one, two, or three asterisks = *p* ≤ 0.05, *p* ≤ 0.01, or *p* ≤ 0.001, respectively.

Af293 strains, naturally infected with AfuPmV-1 (18–95, 10–53), virus-free Af293 (18–42), or 18–42 re-infected with AfuPmV-1 (19–40), were used in the form of conidia (Figure 2A–C), or in the form of hyphae (Figure 2D–F). Fungal growth on SDA plates was compared at 37 °C (normal human body temperature) over 72 h, and colony diameters on agar at each time point were used to calculate areas of growth [mm^2^]. Our results show that the growth of all strains increased over time, whether starting from conidia (Figure 2A–C), or from hyphae (Figure 2D–F). When growth started from hyphae, areas at each timepoint were larger (compare Figure 2A–C to Figure 2D–F). Starting from conidia or from hyphae, the virus free strain 18–42 grew better than any infected strain at each timepoint (Figure 2).

With conidia, these differences in growth at 37 °C were noted in all experiments we performed on agar not containing additives, with inoculum sizes of 10^4^ to 10^7^ conidia/mL. Our data indicate that virus infection interferes with early (started from conidia) fungal growth and extends into later stages of fungal growth (started from hyphae). These differences in growth on agar were also observed for planktonic growth [9,12].

### 3.2. Effect of Heat Stress: Early Growth Advantage of Virus-Free A. fumigatus Af293 Compared to AfuPmV-1-Infected Fungus at 46 °C

**Figure 3:**
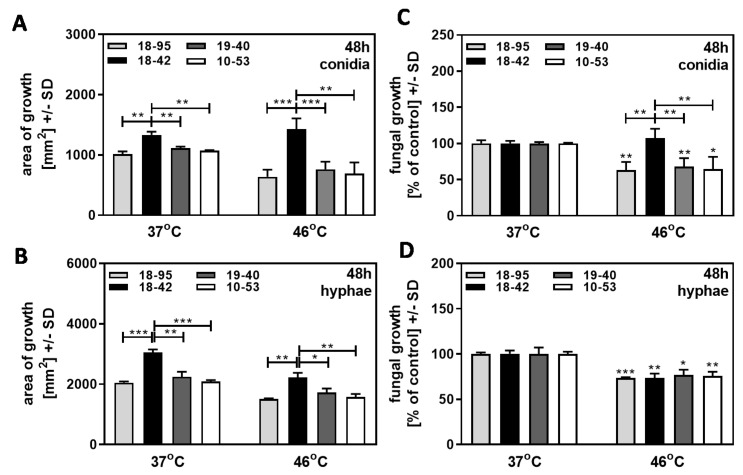
Effect of heat stress: early stage growth advantage of virus-free *A. fumigatus* Af293 over AfuPmV-1-infected fungus at 46 °C. AfuPmV-1-infected *A. fumigatus* Af293 (18–95, 10–53), virus-free Af293 (18–42), and reinfected Af293 (19–40) were placed on SDA plates at 10^4^ conidia/plate (**A**,**B**), or, in the form of hyphae, derived from SDA plates at 48 h of incubation (**C**,**D**) and incubated at 37 °C or 46 °C for 48 h. Areas of growth were determined in mm^2^ (**A**,**B**). Growth at 37 °C for each strain was regarded as 100%, and growth at 46 °C was normalized to that (**C**,**D**). Comparisons without brackets in (**C**,**D**): each strain at 37 °C (=100%) vs. the same strain at 46 °C. Comparisons to 18–42 as indicated by the ends of the brackets. Statistical analysis: unpaired *t*-test, one, two, or three asterisks = *p* ≤ 0.05, *p* ≤ 0.01, or *p* ≤ 0.001, respectively.

We first investigated if infection would alter fungal resistance to heat stress. Virus-free Af293 (18–42), as well as three Af293 strains infected with AfuPmV-1 (18–95, 10–53, 19–40), were grown on SDA plates at 37 °C, or at 46 °C, over 48 h. Growth initiated from conidia was less than growth initiated from hyphae, at 37 °C as well as at 46 °C (compare Figure 3A to Figure 3B). For growth initiated from conidia (Figure 3A) or from hyphae (Figure 3B), the three infected Af293 strains grew less than the virus-free strain 18–42 at 37 °C and at 46 °C. After normalization to the individual strain’s growth at 37 °C, regarded as 100%, the virus-free strain initiating from conidia showed a growth advantage over all three infected strains at 46 °C (Figure 3C). With growth initiated as hyphae, thus excluding the early growth phase, the virus-free strain no longer showed increased resistance to heat (Figure 3D). Our results indicate that virus infection interfered with resistance to heat stress at early stages, i.e. within the initial 48 h of growth. These initial 48 h of growth were not a factor when growth was initiated with hyphae.

### 3.3. At Room Temperature (22 °C), Virus-Free A. fumigatus Af293 Has No Growth Advantage over AfuPmV-1-Infected Fungus

**Figure 4:**
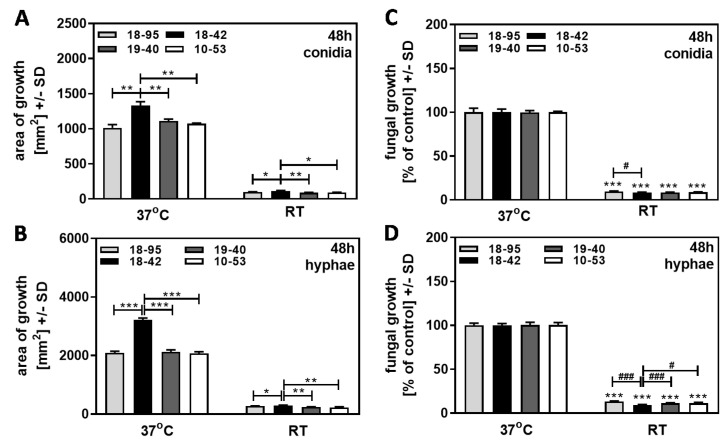
At room temperature, virus-free *A. fumigatus* Af293 has no growth advantage over AfuPmV-1-infected fungus. AfuPmV-1-infected *A. fumigatus* Af293 (18–95, 10–53), virus-free Af293 (18–42), and reinfected Af293 (19–40) were placed on SDA plates at 10^4^ conidia/plate (**A**,**B**), or, in the form of hyphae, derived from SDA plates at 48 h of incubation (**C**,**D**) and incubated at 37 °C or room temperature (22 °C) for 48 h. Areas of growth were determined in mm^2^ (**A**,**B**). Growth at 37 °C for each strain was regarded as 100%, and growth at room temperature was normalized to that (**C**,**D**). Comparisons without brackets in (**C**,**D**): each strain at 37 °C (=100%) vs. the same strain at room temperature. Comparisons to 18–42 as indicated by the ends of the brackets. Statistical analysis: unpaired *t*-test, one, two, or three asterisks or pound signs = *p* ≤ 0.05, *p* ≤ 0.01, or *p* ≤ 0.001, respectively. Asterisks represent decreases in growth; pound signs represent increases in growth.

Virus-free Af293 (18–42), as well as three Af293 strains infected with AfuPmV-1 (18–95, 10–53, 19–40), were grown on SDA plates at 37 °C or at room temperature over 48 h. In part of the experiment, we used conidia as a starting point (Figure 4A,C); in another part of the experiment, we excluded the effects on early growth stages by initiating growth with hyphae (Figure 4B,D). All four Af293 strains grew much less at 22 °C compared to 37 °C, regardless of the infection status (compare Figure 4A to Figure 4B). As already observed in Figure 2, all strains achieved greater total growth, at 37 °C as well as at RT, when growth was initiated from hyphae (Figure 4A vs. Figure 4B). When compared to each strain’s growth at 37 °C, regarded as 100%, the virus-free strain did not have a growth advantage over any of the infected strains, regardless of initiation from conidia (Figure 4C) or from hyphae (Figure 4D). Our results indicate that conidia and hyphae of infected and virus-free fungi were equally affected by growth conditions colder than optimal.

### 3.4. Heating or Freezing of Conidia Does Not Differentially Affect Fungal Growth

**Figure 5:**
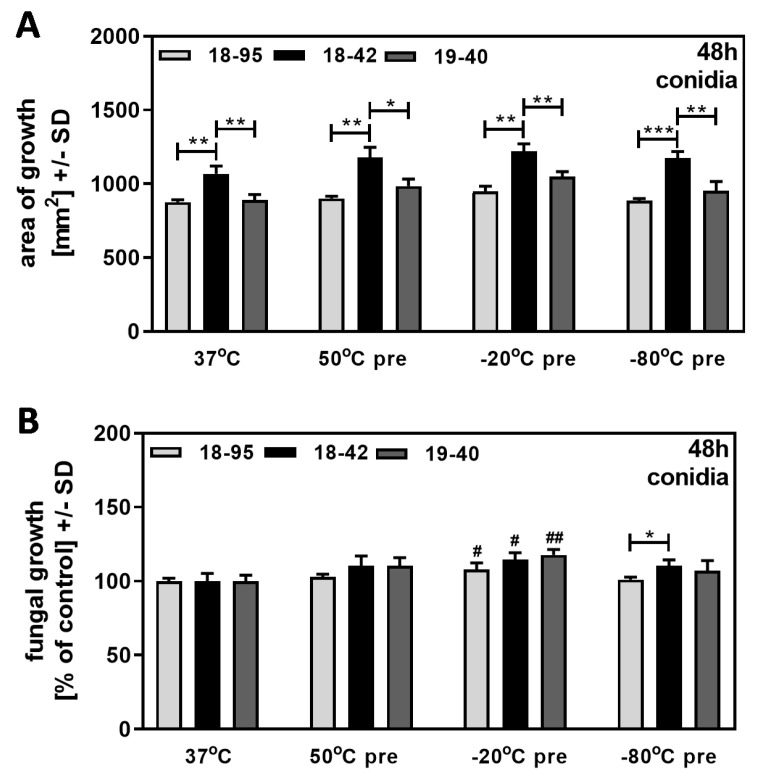
Heating or freezing of conidia does not affect fungal growth. Conidia suspensions of 10^7^/mL for AfuPmV-1-infected *A. fumigatus* Af293 (18–95), virus-free Af293 (18–42), and reinfected Af293 (19–40) were treated by heating at 50 °C for 30 min, freezing at −20 °C or −80 °C for 24 h, or used without pre-incubation. Suspensions were equilibrated to room temperature, placed on SDA plates at 10^4^ conidia/plate, and incubated at 37 °C for 48 h. Diameters of colonies were measured and areas of growth were determined in mm^2^ (**A**). Growth of non-pretreated conidia (=37 °C) for each strain was regarded as 100%, and growth of pre-treated conidia was normalized to that (**B**). Comparisons without brackets in (**B**): control (37 °C) vs. all other bars. Comparisons to 18–42 as indicated by the ends of the brackets. Statistical analysis: unpaired *t*-test, one, two, or three asterisks or pound signs = *p* ≤ 0.05, *p* ≤ 0.01, or *p* ≤ 0.001, respectively. Asterisks represent decreases in growth; pound signs represent increases in growth.

We pre-treated conidia suspensions of 10^7^ conidia/mL by heating to 50 °C for 30 min or by freezing in RPMI medium at −20 °C or −80 °C for 24 h. All suspensions were allowed to equilibrate to room temperature before evaluating growth on SDA plates at 37 °C after 48 h of incubation. Our results show that treatment did not affect the ability of virus-free Af293 (18–42) to grow better than both infected strains (18–95 and 19–40) (Figure 5A). When growth of pretreated fungus was normalized to growth at 37 °C, regarded as 100% for each strain, 18–42 did not show a growth advantage over both infected strains at any condition (Figure 5B).

Our results indicate that heat or cold stress to conidia does not affect superior growth of the virus-free strain on subsequent culture, but there was no advantage relative to control growth, as seen when stress occurred during the growth phase on agar. This suggests that viral impairment of fungal resistance does not extend to heat or cold stress limited to conidia prior to growth.

### 3.5. Congo Red (CR) Inhibits Fungal Growth, with Virus-Free A. fumigatus Af293 Less Susceptible to CR Than AfuPmV-1-Infected Fungus

Growth of AfuPmV-1-infected *A. fumigatus* Af293 (naturally infected strain 18–95), from 10^3^ conidia/plate on SDA plates, was observed over 72 h and showed significant daily increases (Supplemental Figure S1A). When growth on SDA plates for each time point, defined as 100%, was compared to growth on SDA plates containing CR at 100, 200, or 400 µg/mL, we found dose-dependent inhibition (Supplemental Figure S1B). Inhibition was about equal at each time point of observation (Supplemental Figure S1B). For further experiments, we disregarded measurements at 24 h, as growth on SDA plates was minimal (Supplemental Figure S1A). We also decided to use CR at 400 µg/mL for subsequent experiments, the concentration closest to the IC50.

In initial experiments of this study, we used 10^3^ conidia/SDA plate (10 µL of a 10^5^ conidia/mL suspension) to initiate growth (Supplemental Figure S1). We then compared this conidia concentration to concentrations 100× higher or 10× lower but did not find differences in the susceptibility of 18–95 to CR at 48 h (Supplemental Figure S2A) or 72 h (Supplemental Figure S2B). For further experiments, we decided to study 10^2^ conidia/plate (10 µL of a conidia suspension containing 10^4^ conidia/mL).

**Figure 6:**
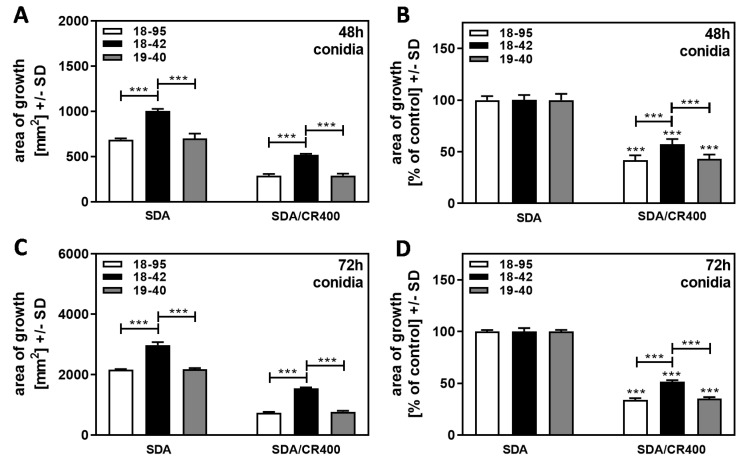
Virus-free Af293 is less susceptible to CR than AfuPmV-1-infected Af293. Naturally AfuPmV-1-infected *A. fumigatus* Af293 (strain number 18–95), cured 18–95 (18–42), and reinfected 18–42 (19–40) were placed on SDA, or on SDA containing 400 µg/mL Congo Red (CR), at 100 conidia/plate, and incubated at 37 °C for 48 (**A**,**B**) or 72 h (**C**,**D**). Areas of growth were calculated in mm^2^ (**A**,**C**). For each timepoint, growth on SDA plates was regarded as 100% and growth on CR-containing plates was normalized to that (**B**,**D**). Comparisons without brackets in (**B**,**D**): each strain without CR (=100%) vs. the same strain with CR. Comparisons to 18–42 as indicated by the ends of the brackets. Statistical analysis: unpaired *t*-test, three asterisks = *p* ≤ 0.001.

When comparing Af293 strains either naturally infected with AfuPmV-1 (18–95), virus-free (18–42), or virus-free but re-infected with AfuPmV-1 (19–40), we found that after 48 h of incubation, colonies of 18–42, started from conidia, on SDA plates and on SDA plates containing CR [400 µg/mL] grew better than both infected strains (Figure 6A). When growth was normalized for each strain’s growth on SDA = 100%, it emphasized that 18–42 tolerated CR significantly better than both infected strains (Figure 6B). Results before (Figure 6C) and after normalization (Figure 6D) were even clearer after 72 h of incubation, and we observed in multiple experiments that CR effects grew stronger after longer incubation (compare Figure 6B to Figure 6D).

**Figure 7:**
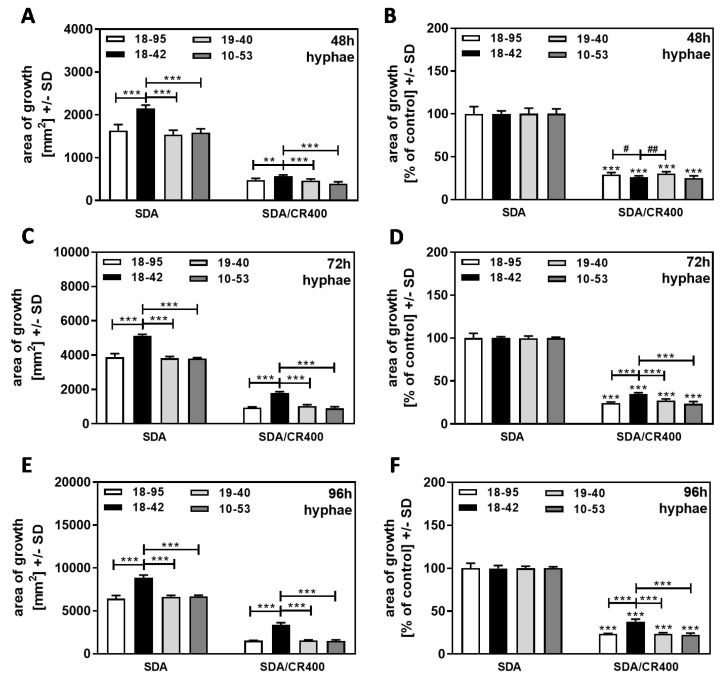
Hyphae-based cultures are susceptible to CR. AfuPmV-1-infected *A. fumigatus* Af293 (18–95, 10–53), virus-free Af293 (18–42), and reinfected Af293 (19–40) were placed on SDA in the form of hyphae derived from SDA plates after 48 h of incubation and then incubated at 37 °C for 48 (**A**,**B**), 72 (**C**,**D**), or 96 h (**E**,**F**). Areas of growth were determined in mm^2^. In (**B**,**D**,**F**), growth without CR for each strain was regarded as 100%, and growth with CR was normalized to that. Comparisons without brackets in (**B**,**D**,**F**): each strain growing without CR (=100%) vs. the same strain growing with CR. Comparisons to 18–42 as indicated by the ends of the brackets. Statistical analysis: unpaired *t*-test, two, or three asterisks or pound signs = *p* ≤ 0.01, or *p* ≤ 0.001, respectively. Asterisks indicate significant decreases; pound signs indicate significant increases.

When the same experiment was performed with cultures that started from hyphae, we found that, again, the virus-free strain 18–42 had a growth advantage over all infected strains at 48, 72, and 96 h (Figure 7A,C,E). After normalization to each strain’s growth on SDA = 100% (Figure 7B,D,F), higher resistance of 18–42 to CR was observed, starting at 72 h (Figure 7D) and becoming more pronounced at 96 h (Figure 7F). Interestingly, at 48 h, no increased resistance of hyphae-derived 18–42 cultures to CR was observed (Figure 7B), whereas conidia-derived 18–42 cultures were more resistant than all infected strains at 48 h (Figure 6B).

### 3.6. Virus-Free Af293 Is Less Susceptible to Hydrogen Peroxide Than AfuPmV-1-Infected Af293 on SDA and RPMI Agar

In our initial experiments, we exposed 10^2^ conidia of virus-infected strain 18–95 to a range of serial H_2_O_2_ concentrations, 0–882 mM, in a 0.5 mL volume for 1 h at 37 °C, then placed the mixtures over agar and counted CFU at 24 h. We found that exposure to ≤32.6 mM did not reduce CFU compared to 0 mM, and that concentrations ≥ 62.6 mM reduced CFU by >92%. A concentration of 42.6 mM reduced CFU by 60%. When conidia of infected strains 18–95 and 19–53 and re-infected strain 19–40 were compared after a 1 h, 42.6 mM, exposure with virus-free strain 18–42, there were small but non-significant differences in CFU at 24 h.

That concentration was used for further experiments (six were conducted). We next tested strains 18–95, 19–53, and 19–40 concurrently with strain 18–42 and measured radial growth over 96 h after plating of an inoculum in the center of the plate. We found that this brief exposure of conidia to H_2_O_2_ (plus some small carryover of H_2_O_2_ onto the plate from the inoculum) inhibited the subsequent growth of all strains; virus-free strain 18–42 growth over 96 h was superior to the infected strains, with or without H_2_O_2_ exposure; and 18–42 growth for the first 48 h after H_2_O_2_ exposure also exceeded that of the infected strains after H_2_O_2_, when growth in H_2_O_2_ was calculated as a fraction of growth in the absence of H_2_O_2_.

We then chose to also examine the effects of continuous H_2_O_2_ exposure during growth by incorporating H_2_O_2_ into the agar before adding conidia (unexposed to H_2_O_2_) to the plates. A dose–response study (10–40 mM) determined that a 30 mM final H_2_O_2_ concentration in agar gave consistent strain inhibition.

**Figure 8:**
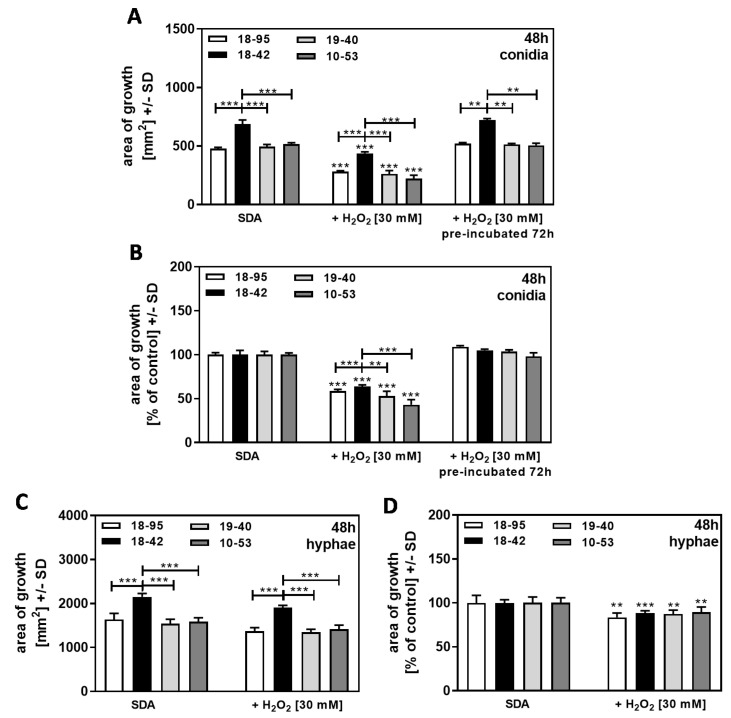
Growth advantage of virus-free *A. fumigatus* Af293 compared to AfuPmV-1-infected fungus in the presence of H_2_O_2_ on SDA. A and B: AfuPmV-1-infected *A. fumigatus* Af293 (18–95, 10–53), virus-free Af293 (18–42), and reinfected Af293 (19–40) conidia were placed on SDA not containing H_2_O_2_ (left four bars in (**A**,**B**)), or containing H_2_O_2_ at 30 mM (the middle quartets of (**A**,**B**) are showing results on freshly prepared agar, and the right quartets are showing results on plates that had been pre-incubated at 37 °C for 72 h before conidia were seeded at 10^4^ conidia/plate). All plates were incubated with conidia at 37 °C for 48 h. (**C**,**D**): AfuPmV-1-infected *A. fumigatus* Af293 (18–95, 10–53), virus-free Af293 (18–42), and reinfected Af293 (19–40) hyphae were derived from SDA plates after 48 h of incubation, and hyphae-based colonies were investigated for growth on RPMI plates at 48 h. Diameters of colonies were measured, and areas of growth were determined in mm^2^ (**A**,**C**). Growth without H_2_O_2_ for each strain was regarded as 100%, and growth with H_2_O_2_ was normalized to that (**B**,**D**). Comparisons without brackets: each strain without H_2_O_2_ vs. the same strain with H_2_O_2_. Other comparisons as indicated by the ends of the brackets for 18–42 vs. all other strains. Statistical analysis: unpaired *t*-test, two, or three asterisks = *p* ≤ 0.01, or *p* ≤ 0.001, respectively.

Af293 strains, either naturally infected with AfuPmV-1 (18–95, 10–53), virus-free (18–42), or virus-free but re-infected with AfuPmV-1 (19–40), were exposed to H_2_O_2_ during growth to induce continuous oxidative stress. We found that after 48 h of incubation, conidia-started colonies of 18–42 on SDA plates and on SDA plates containing H_2_O_2_ [30 mM] grew better than all infected strains (Figure 8A, left quartet and middle quartet). After normalization to each strain’s growth on SDA = 100%, a higher resistance of 18–42 to H_2_O_2_, compared to all infected strains, was observed (Figure 8B, left quartet and middle quartet).

When SDA + H_2_O_2_ agar were pre-incubated at 37 °C for 72 h before conidia were added, no growth inhibition (Figure 8A, right and left quartets), and no growth advantage of 18–42 over infected strains (Figure 8B, right and left quartets) was observed, indicating that H_2_O_2_ in plates loses activity over time.

When cultures were started from hyphae, we found 18–42 growing better than all infected strains on SDA agar, with fresh H_2_O_2_ or without H_2_O_2_ present (Figure 8C). After normalizing to each strain’s growth on SDA = 100%, no higher resistance of 18–42 to H_2_O_2_ was observed compared to all infected strains (Figure 8D). This indicates that the effect of H_2_O_2_ is on the phase of growth when conidia germinate and grow as hyphae.

**Figure 9:**
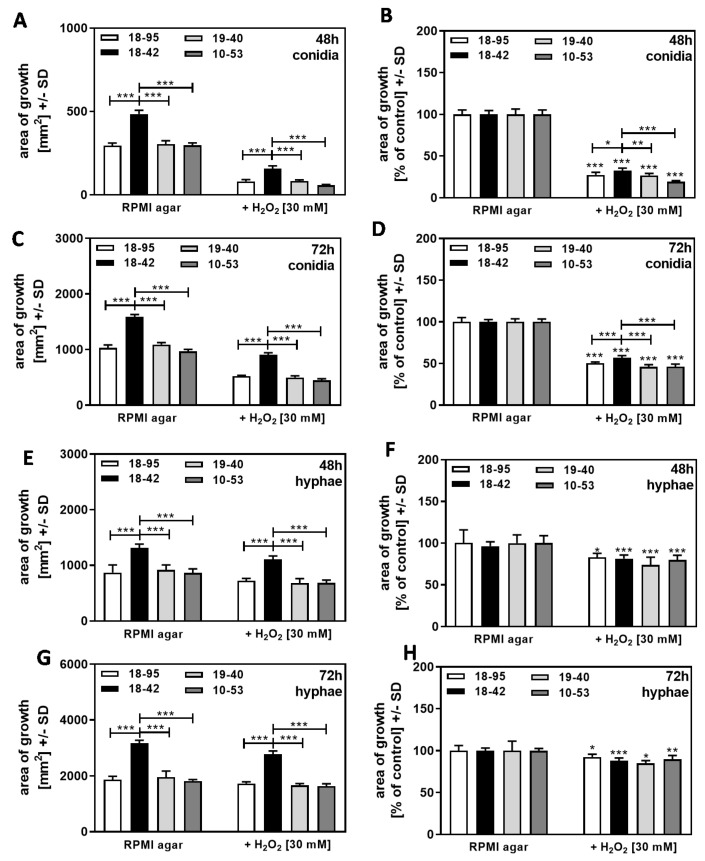
Growth advantage of virus-free *A. fumigatus* Af293, compared to AfuPmV-1-infected fungus in the presence of H_2_O_2_ on RPMI agar. **A**–**D**: AfuPmV-1-infected *A. fumigatus* Af293 (18–95, 10–53), virus-free Af293 (18–42), and reinfected Af293 (19–40) conidia were placed on RPMI agar plates not containing H_2_O_2_ or containing H_2_O_2_ at 30 mM and incubated at 37 °C for 48 h (**A**,**B**) or 72 h (**C**,**D**). (**E**–**H**): AfuPmV-1-infected *A. fumigatus* Af293 (18–95, 10–53), virus-free Af293 (18–42), and reinfected Af293 (19–40) hyphae were derived from SDA plates after 48 h of incubation, reinoculated on RPMI agar plates, and the growth of the resultant colonies was assessed after 48 h (**E**,**F**) or 72 h (**G**,**H**) of incubation. Diameters of colonies were measured, and areas of growth were determined in mm^2^ (**A**,**C**,**E**,**G**). Growth without H_2_O_2_ for each strain was regarded as 100%, and growth with H_2_O_2_ was normalized to that (**B**,**D**,**F**,**H**). Comparisons without brackets: each strain without H_2_O_2_ vs. the same strain with H_2_O_2_. Other comparisons as indicated by the ends of the brackets for 18–42 vs. all other strains. Statistical analysis: unpaired *t*-test, one, two, or three asterisks = *p* ≤ 0.05, *p* ≤ 0.01, or *p* ≤ 0.001, respectively.

The experiments on SDA were repeated on RPMI agar, a defined medium that provides less nutrients and iron than SDA. Results obtained on RPMI agar again showed increased growth of the virus-free strain (Figure 9A,C) and increased resistance of the virus-free strain 18–42 to H_2_O_2_ in cultures that started from conidia at 48 h (Figure 9B) and at 72 h of incubation (Figure 9D). In cultures on RPMI agar that started from hyphae, the growth of the virus-free strain again exceeded the growth of all infected strains (Figure 9E,G), but the virus-free strain did not show higher resistance to H_2_O_2_, relative to control growth, than the infected strains at 48 h (Figure 9F) or 72 h of incubation (Figure 9H).

### 3.7. Agar Content as a Stress Factor

**Figure 10:**
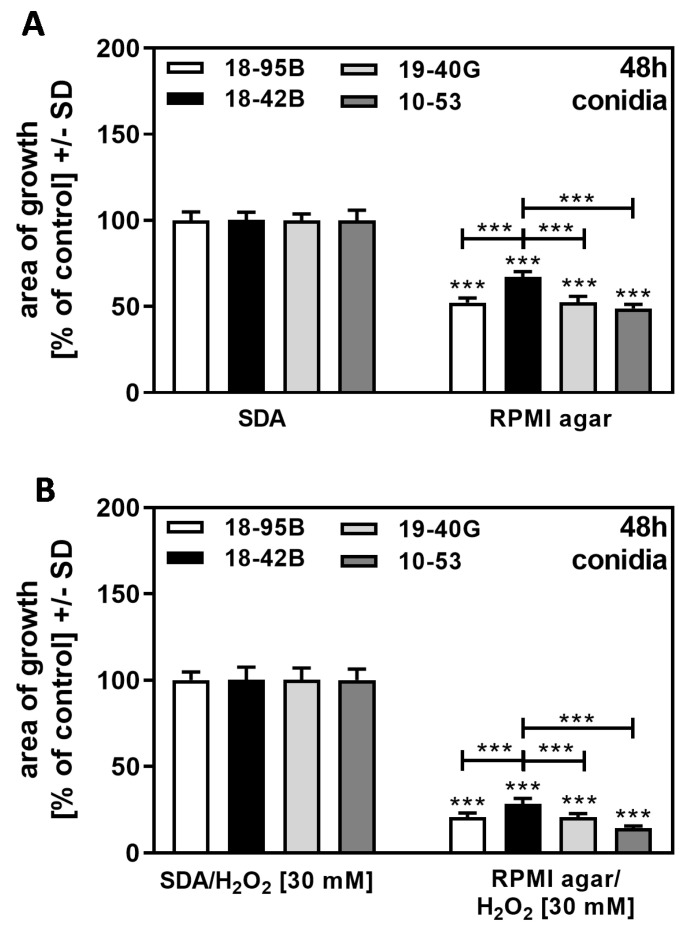
Growth advantage of virus-free *A. fumigatus* Af293 over AfuPmV-1-infected fungus under “starvation stress”. AfuPmV-1-infected *A. fumigatus* Af293 (18–95, 10–53), virus-free Af293 (18–42), and reinfected Af293 (19–40) conidia were placed on SDA and RPMI agar plates not containing H_2_O_2_ (**A**) or containing H_2_O_2_ at 30 mM (**B**). All plates were incubated with conidia at 37 °C for 48 h. Diameters of colonies were measured, and areas of growth were determined in mm^2^. Growth on SDA (**A**) or SDA + H_2_O_2_ (**B**) for each strain was regarded as 100%, and growth on RPMI agar (**A**), or RPMI agar + H_2_O_2_ (**B**) was normalized to that. Comparisons without brackets: each strain on SDA vs. the same strain on RPMI agar. Other comparisons as indicated by the ends of the brackets for 18–42 vs. all other strains. Statistical analysis: unpaired *t*-test, three asterisks = *p* ≤ 0.001.

Fungal growth on RPMI agar was less compared to SDA (compare Figure 9A,C,E,G to Figure 8A,C). When we normalized growth on RPMI agar to growth on SDA, we found the virus-free strain 18–42 to be more resistant than all infected strains (Figure 10A). The same result was obtained when RPMI agar + H_2_O_2_ was normalized to SDA + H_2_O_2_ = 100% for each strain (Figure 10B). Our results indicate that growth on RPMI agar provided more stress (“starvation stress”) for infected strains than for the virus-free strain.

## 4. Discussion

We have shown AfuPmV-1-infected Af293 to be more sensitive to a variety of stresses compared to virus-free Af293, including bacterial filtrates [8] and volatiles [8], high salt [11], and nikkomycin Z (NikZ) [12], but not amphotericin B (AmB), voriconazole (VCZ), or micafungin (MICA) [12]. The impairment of the stress response to bacterial filtrates relates to a delay of siderophore release in the competition of iron [9]. Here, we show that viral impairment of the stress response is quite broad. AfuPmV-1-infected Af293 is also more sensitive to other stresses: high temperature, CR treatment, exposure to hydrogen peroxide, and nutrient restriction. Similarly, Af293 infected with the variant AfuPmV-1M was reported to decrease tolerance to osmotic stress caused by high salt and oxidative stress caused by hydrogen peroxide [7]. The former observation was attributed to the proteins encoded by dsRNAs 2 and 5, respectively a putative scaffold protein and the protein present in AfuPmV-1M, but not AfuPmV-1, while the latter to the putative scaffold protein and the methyl transferase encoded by dsRNA 3. In addition to *Polymycoviridae*, infection of *A. fumigatus* with a member of *Chrysoviridae* resulted in sensitivity to osmotic and oxidative stresses, but also impaired the host in the presence of formic acid and nitric oxide and under hypoxic low oxygen conditions [13]. In *A. flavus*, infection with a member of *Partitiviridae* also weakened the host against osmotic, oxidative, and ultraviolet stress [14].

We found here that treatment of conidia-based cultures with high temperatures revealed a clear growth disadvantage for infected fungal cultures, whereas no relative differences were observed when conidia were only pre-treated with high or low temperatures, when conidia grew at room temperature, or when hyphae-based cultures were treated with high temperatures. It seems that high temperature only affects infected *A. fumigatus* more strongly when the fungus is in its developmental growth phase, but not when it is exposed short-term in its conidial form, or exposed once it reached its hyphal form. During growth, it is feasible that high temperature damages essential enzymes and DNA, and may induce oxidative stress to the fungus [15].

Oxidative stress can induce damage and even apoptosis in pathogenic fungi [16]. This can be especially problematic in certain cellular organelles like mitochondria, providing power to cells, where high temperature can cause dysfunction or even destruction [17,18,19]. It has been shown that the heat shock protein *hsfA* regulates the expression of genes involved in heat shock response, cell wall biosynthesis and remodeling, and lipid homeostasis [20], all being most essential during fungal growth. Fungal response to high temperatures, as well as to other environmental stresses, also is regulated through the high-osmolarity glycerol mitogen-activated protein kinase (HOG-MAPK) pathway [21]. It is known that *A. fumigatus*, in response to oxidative and high-temperature stress, exposure to antifungal compounds like thymol, farnesol, citral, nerol, and salicylic acid, and exposure to bacterial compounds like phenazine-1-carbonic acid, and pyocyanin, generates reactive oxygen and nitric oxygen species [22]. A study in *Cryphonectria parasitica*, a phytopathogenic fungus that causes chestnut blight, showed that infection with the mycovirus Cryphonectria hypovirus 1 (CHV1) induces oxidative stress [23]. Assuming that infection with the mycovirus AfuPmV-1 also induces oxidative stress, or impairs responses to oxidative stress, this would explain a growth advantage of virus-free fungus compared to infected fungus, as we here observed under different growth conditions and on different agars. Adding more oxidative stress, using high temperature, or the classic inducer of oxidative stress, hydrogen peroxide, affected infected fungus more.

We found that hydrogen peroxide in agar plates was most active when freshly prepared, and lost activity within 72 h of incubation. Fungal growth, especially on RPMI agar plates, was very weak after 24 h of incubating conidia, not allowing reliable measurement of culture growth before 48 h of incubation. As hydrogen peroxide activity declined rapidly at 37 °C, differences in infected and virus-free strain resistance to hydrogen peroxide were weaker compared to other stresses used in the present study, but still significant.

Effects of hydrogen peroxide were more pronounced on conidia-based cultures compared to hyphae-based cultures. Starting from hyphae-based cultures, we did not observe a growth advantage of virus-free fungus compared to infected fungus under hydrogen peroxide exposure, when growth was normalized to non-hydrogen peroxide conditions. This observation corresponds to our findings, comparing normalized growth at 37 °C to 46 °C. It seems that oxidative stress, caused by hydrogen peroxide or temperature, had its highest impact on the early growth phase of fungus, when conidia developed into hyphae, but was absent or diminished when hyphae already had formed. We also performed a series of experiments where conidia were exposed to hydrogen peroxide for one hour before seeding on agar not containing hydrogen peroxide. Results were similar to those obtained when hydrogen peroxide was added to agar, but differences between strains were weaker (data not shown), confirming that early events in fungal growth are most affected by oxidative stress, and that viral impairment of conidial viability is not prominent.

When performing hydrogen peroxide experiments on SDA and RPMI agar, we noticed reduced growth on RPMI agar, likely caused by a relative lack of nutrients in RPMI agar. The virus-free strain showed increased resistance to those ‘starvation stress’ conditions. In previous studies, we found that the virus-free *Aspergillus* strain is more resistant to anti-fungal effects of *P. aeruginosa* filtrates, especially to the *Pseudomonas* siderophore pyoverdine [8]. It therefore is feasible that a lack of iron in RPMI agar caused impaired growth of all Af293 strains, and that the virus-free strain tolerated low iron conditions on RPMI agar better than the infected strains. Iron-deficiency is also a known channel for oxidative stress, likely owing to the iron dependence of key enzymes in mitigating oxidative stress.

Our third stressor, Congo Red (CR), like MICA or caspofungin (CASPO), is an inhibitor of beta 1,3 glucan synthase, an enzyme that is crucial for cell wall synthesis [24]. In a previous study, we found that MICA and CASPO similarly affected virus-free and infected fungus [12], indicating that the ability of CR to inhibit beta 1,3 glucan synthase might not be causal for its reduced effects on virus-free fungus. The drug to which we found infected fungus to be more susceptible than virus-free fungus, NikZ [12], is an inhibitor of chitin synthase. CR has high affinity for chitin [25,26] and increases chitin microfibril assembly [27]. CR also has been shown to enhance chitin synthase activity in yeast [28]. Additionally, CR enhances chitin synthesis in growing cells of *Geotrichum lactis* [29]. It is likely that the effects on chitin synthase in fungi are an escape mechanism for the fungus when cell wall synthesis of beta glucan is blocked [30]. These activities essentially are the opposite of the action of NikZ, so it seems unlikely that opposing effects on the same enzyme would lead to the same stronger anti-fungal effect on infected fungus compared to virus-free fungus. There also is the possibility that CR, like high temperature, and hydrogen peroxide, induces oxidative stress in fungi. CR is an azo dye, and azo dyes have been shown to cause oxidative stress in various cell types and animal models [31,32,33,34,35]. This is thought to be caused by their ability to generate ROS when exposed to light, or in the presence of reducing agents [36]. Studies in the brain showed that CR, used to visualize amyloid plaques as signs of neurodegenerative diseases, increases the toxicity of amyloid deposits, which may be caused by increasing oxidative stress [37,38].

In contrast to effects observed for high temperature and hydrogen peroxide stress, CR effects on *A. fumigatus* did not subside with incubation time, suggesting that CR is stable in agar. We even observed more overall growth reduction of fungus after longer incubation, and especially in hyphae-based cultures, in contrast to our other stressors. We speculate that CR, or a metabolite, accumulates in hyphae, suppressing growth. Growth suppression was more pronounced in the infected fungus, leading to a larger difference in growth after normalization to cultures growing without CR. This may suggest viral impairment of the response to CR during later stages of hyphal development, compared to impairment with the other stresses.

Taken together, our three stressors, temperature, hydrogen peroxide, and CR, as a common denominator, could cause oxidative stress for the fungus. Infection with AfuPmV-1 might cause oxidative stress as well, similar to observations made for the mycovirus CHV1 [23], and added oxidative stress might therefore differentially affect infected fungi. More recently, Takahashi-Nakaguchi et al. [7] showed that AfuPmV-1M infection leads to repression of genes involved in the defense of *A. fumigatus* against reactive oxygen species.

Although AmB has been shown to induce oxidative stress in *A. fumigatus* [39], we did not observe significant differential effects of AmB on infected compared to virus-free fungus [12]. The reason might be the quantity of drug-induced oxidative stress on the fungus; if added oxidative stress is low, higher relative resistance of the virus-free strain might not be detectable. Oxidative stress may be a minor component of its anti-fungal action, as AmB’s principal action relates to its effect on membrane pores [40].

We now have a more complete picture of the global nature of the impairment of the fungal host owing to this viral infection. This impairment may explain why surveys of *A. fumigatus* in nature show a minority infected with this virus [4], as the virus-infected cohort may be diminished by environmental stresses, depending on the nature and amounts of stresses encountered in various niches. Our present research efforts are directed to assessing expression of the fungal genes that are understood to underly the responses to the stresses described.

Infections with other fungal viruses enhance their survival in some environments [2]. That AfuPmV-1 decreases *A. fumigatus* ability to cope with stresses suggests that research developments to make the virus more infectious for the fungus could lead to environmental mitigation in nature, decreasing, for example the adverse impact of the fungus on agriculture, on the food supply, in building contamination and decay, on allergies, and on nosocomial infections and opportunistic infections. Such strategy has been successfully employed with other fungal viruses [41], and viral (phage) infection of bacteria is under study as a therapeutic strategy against bacterial infections [42,43].

## Figures and Tables

**Table 1:** *A. fumigatus* strains used in this study.

Strain	CIMR Number	Origin	AfuPmV-1 Infection	Ref.
Af293	10–53	USA	Naturally infected	[1]
Af293	18–95	UK	Naturally infected	[1]
Af293	18–42	UK	18–95 cured of infection	[3]
Af293	19–40	UK	18–42 cured and re-infected	[3]

## Data Availability

Data supporting reported results are available from the corresponding author.

## Supplementary Materials

The following supporting information can be downloaded at: https://www.mdpi.com/article/10.3390/jof9070750/s1, Figure S1: Congo Red inhibits growth of AfuPmV-1-infected A. fumigatus Af293 dose-dependently; Figure S2: Congo Red inhibits growth of AfuPmV-1-infected A. fumigatus Af293 at a wide range of conidia concentrations.
