# Supplementary figures and images for: AfuPmV-1-Infected Aspergillus fumigatus Is More Susceptible to Stress Than Virus-Free Fungus
Source: J Fungi (Basel). 2023 Jul 15;9(7):750. doi: 10.3390/jof9070750 (PMC10381315; doi:10.3390/jof9070750)

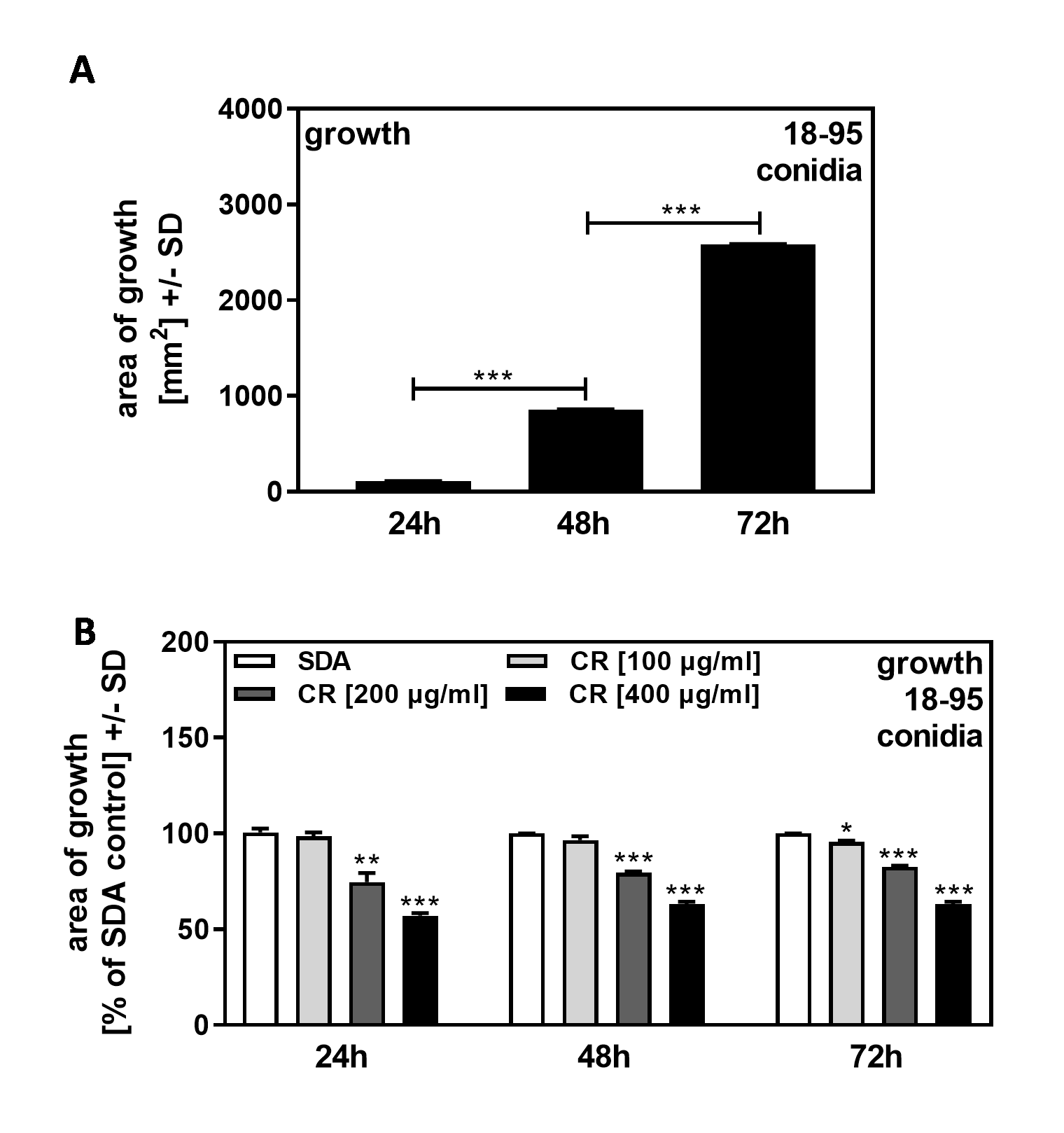

Supplement: Supplementary file 1 [file jof-09-00750-s001.zip › Supplemental Figure 1.tif]

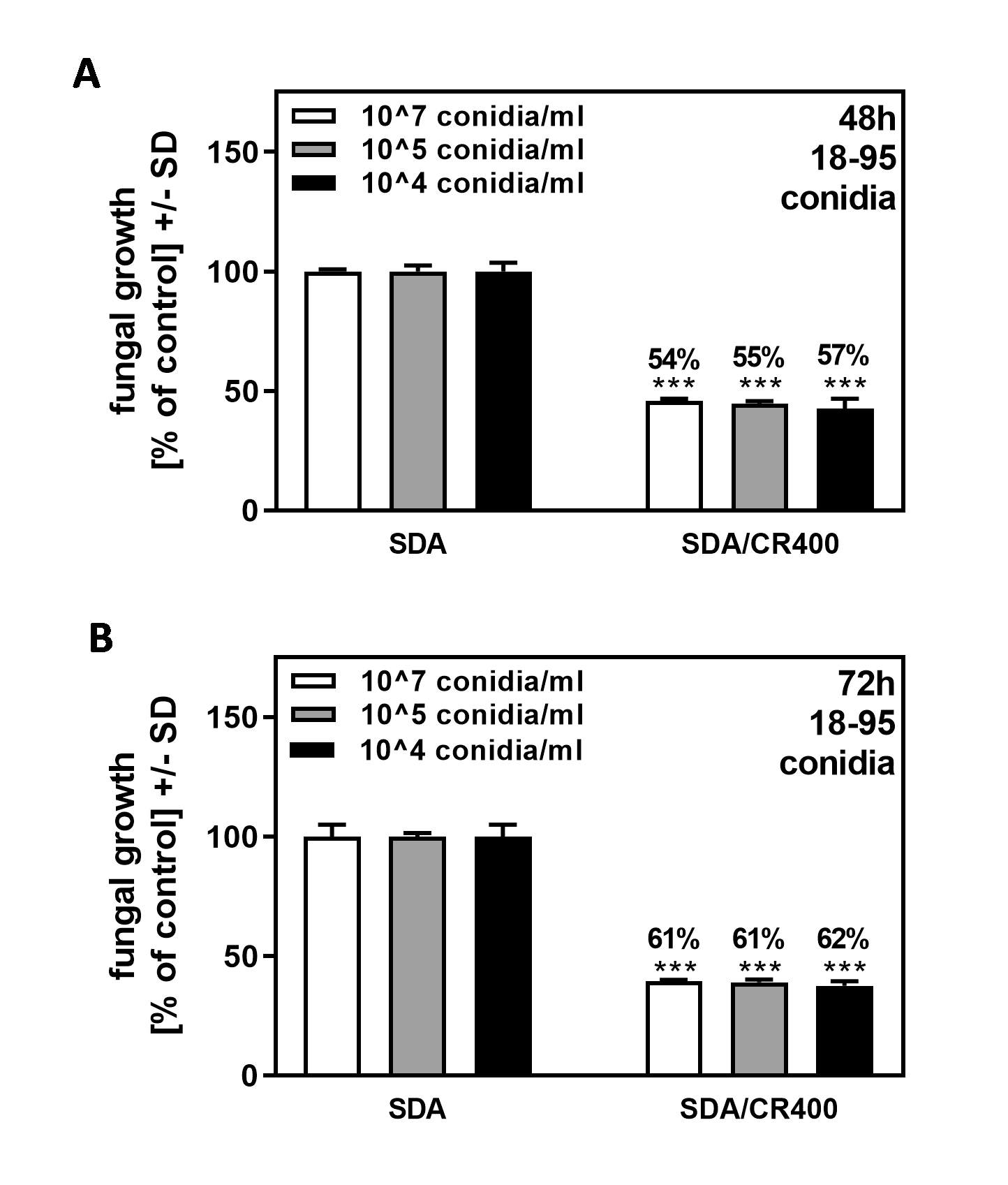

Supplement: Supplementary file 1 [file jof-09-00750-s001.zip › Supplemental Figure 2.tif]
